# Where Shall the American Dental Association Meet in 1886?

**Published:** 1886-04

**Authors:** Chas. S. Tee


### Where Shall the American Dental Association Meet in 1886.

The following communications give some information on the subject, to say the least. The following certainly extends a very hearty invitation to meet in San Francisco:

*Dr. Wm. N. Morrison of Ex. Com. of Amer. Dental Association:*

DEAR SIR:—At a meeting of the San Francisco Dental Association, held in this city on Monday, March 8th, the committee appointed on February 15th to sound the profession as to their wish to have this distinguished body which you represent meet here at their next session, presented such flattering testimonials as to their desire to have you do so, and proffered such generous aid to give you a hearty welcome, that the following resoluion was passed by a unanimous vote: "Resolved that the American Dental Association be cordially invited to hold their coming session in San Francisco." Please let us know as soon as possible the decision of your committee, so that if we are to have the honor as well as the pleasure of your presence here we can commence at once to arrange for your lodgment and comfort, etc., also as to exact time that you will arrive and take your departure. We would suggest that the session be held either prior to or just after the meeting of the Grand Army people, who are to assemble here on August 2d, as the hotel and other accommodations will then be infinitely better. Trusting you will accept our invitation, for we assure you it comes from our hearts, we are most sincerely and fraternally yours,

Committee, THOS. MORFFEW,

W. J. YOUNGER,

WM DUTCH,

S. E. KNOWLES.

Dr. S. W. Dennis, Dean of Faculty, Dental Department University of California, and President of the Board of Examiners, telegraphs, "You will be received with open arms by dental societies and the people if you accept an invitation. Come!"

---

The following pretty clearly indicates the arrangements that can be made with the railroads:

The Union and Central Pacific Railways have guaranteed to Dr. Dudley for the American Dental Association, its members, delegates, and all dentists and their families desiring to attend the meeting, a rate of $50.00 dollars for round trip from the Missouri River to San Francisco and return, going by the above route and returning by any other.

The Grand Army of the Republic have selected August 3d, 1886, at San Francisco, as a proper time to hold their National Encampment, also Woman's Relief Corps and Army of the Potomac. The Knights Templars selected August, 1883, as a proper time for holding their conclave at San Francisco.

August is the fruit and flower season, and the time when the Pacific Coast is at its best, and the occasion is one which will never be duplicated in this country.

Should the Association conclude to go to San Francisco to hold their convention of 1886, we can assure all concerned that the the railway lines west of the Missouri River will do all in their power to make their trip and visit to the Pacific Coast as pleasant as it can be made.

The Dental Association, if members enough go, will be run through on a special train, such stops being made as will give them the best views of the country through which they travel, and accompanied by employes of our road, who will do everything to assist in making their trip pleasant.

M. T. Dennis, N. E. Agt., Boston, Mass.
J. W. Morse, Gen'l. Pass. Agt., Omaha, Nebraska.

---

St. Paul, Minn., March 3d, 1886.

*Dr. A. M. Dudley, Salem, Mass:*

Dear Sir—In referring to our conversation with reference to your proposed excursion to San Francisco, also to letters that have passed between us, I beg to state that we will be a party to a $50.00 round trip rate from the Missouri River to San Francisco.

The Union & Central Pacific stand ready to accept this rate from members of your Association on the *west-bound trip*, and we will accept the business on the *return trip* on the regular percentages established between the lines doing the business.

Yours truly, CHAS. S. TEE, G. P. & T. A.

